# Loss of *vhl* in the zebrafish pronephros recapitulates early stages of human clear cell renal cell carcinoma

**DOI:** 10.1242/dmm.024380

**Published:** 2016-08-01

**Authors:** Haley R. Noonan, Ana M. Metelo, Caramai N. Kamei, Randall T. Peterson, Iain A. Drummond, Othon Iliopoulos

**Affiliations:** 1 Center for Cancer Research, Massachusetts General Hospital, Boston, MA 02114, USA; 2 Department of Medicine, Harvard Medical School, Boston, MA 02115, USA; 3 Department of Life Sciences, Faculty of Sciences and Technology, University of Coimbra, Coimbra 3001-401, Portugal; 4 Division of Nephrology, Department of Medicine, Massachusetts General Hospital, Boston, MA 02114, USA; 5 Department of Genetics, Harvard Medical School, Boston, MA 02115, USA; 6 Cardiovascular Research Center, Department of Medicine, Massachusetts General Hospital, Boston, MA 02114, USA; 7 Broad Institute of Harvard and MIT, Cambridge, MA 02114, USA; 8 Division of Hematology-Oncology, Department of Medicine, Massachusetts General Hospital, Boston, MA 02142, USA

**Keywords:** VHL disease, HIF2a, Renal cell carcinoma, Zebrafish cancer model, Pronephros

## Abstract

Patients with von Hippel–Lindau (VHL) disease harbor a germline mutation in the *VHL* gene leading to the development of several tumor types including clear cell renal cell carcinoma (ccRCC). In addition, the *VHL* gene is inactivated in over 90% of sporadic ccRCC cases. ‘Clear cell’ tumors contain large, proliferating cells with ‘clear cytoplasm’, and a reduced number of cilia. *VHL* inactivation leads to the stabilization of hypoxia inducible factors 1a and 2a [HIF1a and HIF2a (HIF2a is also known as EPAS1)] with consequent up-regulation of specific target genes involved in cell proliferation, angiogenesis and erythropoiesis. A zebrafish model with a homozygous inactivation in the *VHL* gene (*vhl^−/−^*) recapitulates several aspects of the human disease, including development of highly vascular lesions in the brain and the retina and erythrocytosis. Here, we characterize for the first time the epithelial abnormalities present in the kidney of the *vhl^−/−^* zebrafish larvae as a first step in building a model of ccRCC in zebrafish. Our data show that the *vhl^−/−^* zebrafish kidney is characterized by an increased tubule diameter, disorganized cilia, the dramatic formation of cytoplasmic lipid vesicles, glycogen accumulation, aberrant cell proliferation and abnormal apoptosis. This phenotype of the *vhl^−/−^* pronephros is reminiscent of clear cell histology, indicating that the *vhl^−/−^* mutant zebrafish might serve as a model of early stage RCC. Treatment of *vhl^−/−^* zebrafish embryos with a small-molecule HIF2a inhibitor rescued the pronephric abnormalities, underscoring the value of the zebrafish model in drug discovery for treatment of VHL disease and ccRCC.

## Abstract (precis)

**Summary:** Zebrafish with an inactivating mutation in the *vhl* gene can be used as a model of early stage clear cell renal cell carcinoma, with applications for genetic studies and drug screens.

## INTRODUCTION

Patients with a germline mutation in the *VHL* gene develop clear cell renal cell carcinoma (ccRCC), retinal and central nervous system hemangioblastomas, pheochromocytomas, pancreatic neuroendocrine tumors, cystadenomas of the pancreas and middle ear, and erythrocytosis (Maher and Kaelin, 1997). In addition to hereditary VHL disease, inactivation of the *VHL* tumor suppressor gene is the earliest molecular lesion identified in more than 90% of sporadic ccRCC (The Cancer Genome Atlas Research Network, 2013).

Kidney tumors are categorized into different subtypes based on their histology. Clear cell is the most prevalent of renal cell tumors (75% of sporadic cases), followed by papillary type I and II tumors (15%), chromophobe tumors (5%) and oncocytomas (5%) (Iliopoulos, 2006; Truong and Shen, 2011). In contrast to patients with sporadic RCC, VHL patients solely develop ccRCC (Chan-Smutko and Iliopoulos, 2010). ‘Clear cell’ renal neoplasms are characterized by the presence of large cells with clear cytoplasm, due to extensive empty vacuoles regarded as fixation artifacts of lipid or glycogen droplets contained in the original tumor (Shuch et al., 2015; Truong and Shen, 2011). ccRCC cells are also characterized by an increased mitotic index and a reduced number of cilia (Schraml et al., 2009).

The VHL protein (pVHL) is a member of an E3 ubiquitin ligase complex that targets the regulatory subunits of hypoxia inducible factors 1a and 2a [HIF1a and HIF2a (HIF2a is also known as EPAS1)] for proteasomal degradation (Maxwell et al., 1999). Inactivation of HIF2a by the VHL tumor suppressor protein is necessary and sufficient for the tumor suppressor function of VHL in ccRCC (Kondo et al., 2002, 2003; Zimmer et al., 2004). HIF1a and HIF2a are expressed in most human epithelial cells and exhibit both distinct and opposing functions (Hu et al., 2003; Raval et al., 2005). In the case of VHL-associated and sporadic ccRCC, HIF2a appears to function as an oncogene and HIF1a as a tumor suppressor gene (Maranchie et al., 2002; Shen et al., 2011).

Inactivation of the *VHL* tumor suppressor gene is the earliest molecular event occurring in the premalignant cystic lesions of VHL patients and in sporadic ccRCC (Lubensky et al., 1996). Nevertheless, it appears that additional mutations are required for the renal epithelial cell to develop a fully malignant phenotype (The Cancer Genome Atlas Research Network, 2013). Inactivation of *Vhl* in the mouse kidney epithelium leads to cellular proliferation, lipid accumulation and cortical cyst formation but not to the formation of ccRCC (Rankin et al., 2006). Comprehensive genomic analysis of human sporadic ccRCC tumors identified recurrent mutations in the PI3K-mTOR pathway, chromatin remodelers and chromatin modifiers, in addition to inactivation of the *VHL* gene (The Cancer Genome Atlas Research Network, 2013).

Zebrafish larvae in which *vhl* has been inactivated (*vhl^−/−^*) recapitulate many aspects of human VHL disease. It has been previously reported that *vhl^−/−^* larvae develop abnormal vascular proliferative lesions in the brain and retina, resembling human hemangioblastomas, as well as erythrocytosis (Van Rooijen et al., 2009, 2010; Metelo et al., 2015). Here, we characterize the epithelial abnormalities present in the kidney of *vhl^−/−^* zebrafish larvae as a first step in building a model of ccRCC in zebrafish. Considering that the hypoxia, angiogenesis and erythropoiesis pathways are conserved between humans and fish, the zebrafish serves as an excellent model to study VHL-associated tumor biology (Kajimura et al., 2006; Rojas et al., 2007; Paffett-Lugassy et al., 2007). In addition, the zebrafish kidney is an appropriate model of kidney development and function, as well as tubule segmentation, because it is simple and composed of the same cell types as in mammals. The genes involved are also conserved. The zebrafish kidney tubule consists of a proximal and a distal segment (Drummond and Davidson, 2010).

There is currently no treatment for VHL disease (Lonser et al., 2003). Patients typically undergo repeated surgeries for multiple tumors that develop over their lifetime. Sporadic ccRCC is commonly treated with inhibitors of the vascular endothelial growth factor (VEGF) signaling pathway, which results in a prolongation of disease progression and overall survival by only a few months (Rini and Atkins, 2009). The limited success of VEGF inhibitors and the subsequent development of resistance is not surprising; VEGF is only one of the many downstream targets of the oncogenic HIF2a. We previously reported the use of a small-molecule HIF2a inhibitor, Compound 76, in the homozygous *vhl* zebrafish model (Zimmer et al., 2008; Metelo et al., 2015). This inhibitor was shown to rescue many of the aspects of VHL disease recapitulated in the zebrafish model, including erythrocytosis, irregular angiogenesis in the brain and retina resembling hemangioblastomas, cardiomegaly with decreased cardiac contractility, abnormal hematopoiesis and early lethality (Metelo et al., 2015). Establishing a zebrafish model for ccRCC and VHL disease might expedite the discovery of novel treatments for VHL-associated tumors.

Here, we show that the proximal *vhl^−/−^* pronephric tubule exhibits structural abnormalities when compared to wild-type (*wt*) siblings (*vhl*^+/+^ and *vhl*^+/−^). These *vhl^−/−^* kidney structures have an augmented tubule diameter, fewer epithelial cells (seen in transverse sections), disorganized cilia, prominent cytoplasmic lipid vesicles, increased glycogen content, aberrant cell proliferation and apoptosis. The overall histological disorganization of the *vhl^−/−^* pronephros, in addition to the presence of cytoplasmic lipid vesicles and glycogen, is reminiscent of ‘clear cell’ histology, indicating that the *vhl^−/−^* mutant zebrafish might serve as a model of early stage ccRCC. In addition, treatment of *vhl^−/−^* zebrafish embryos with the specific HIF2a inhibitor Compound 76 significantly attenuated the pronephric phenotype, demonstrating that the phenotype is HIF2a driven and highlighting the value of zebrafish models for the discovery of novel drugs targeting VHL disease and ccRCC.

## RESULTS

**Fig. 1.**
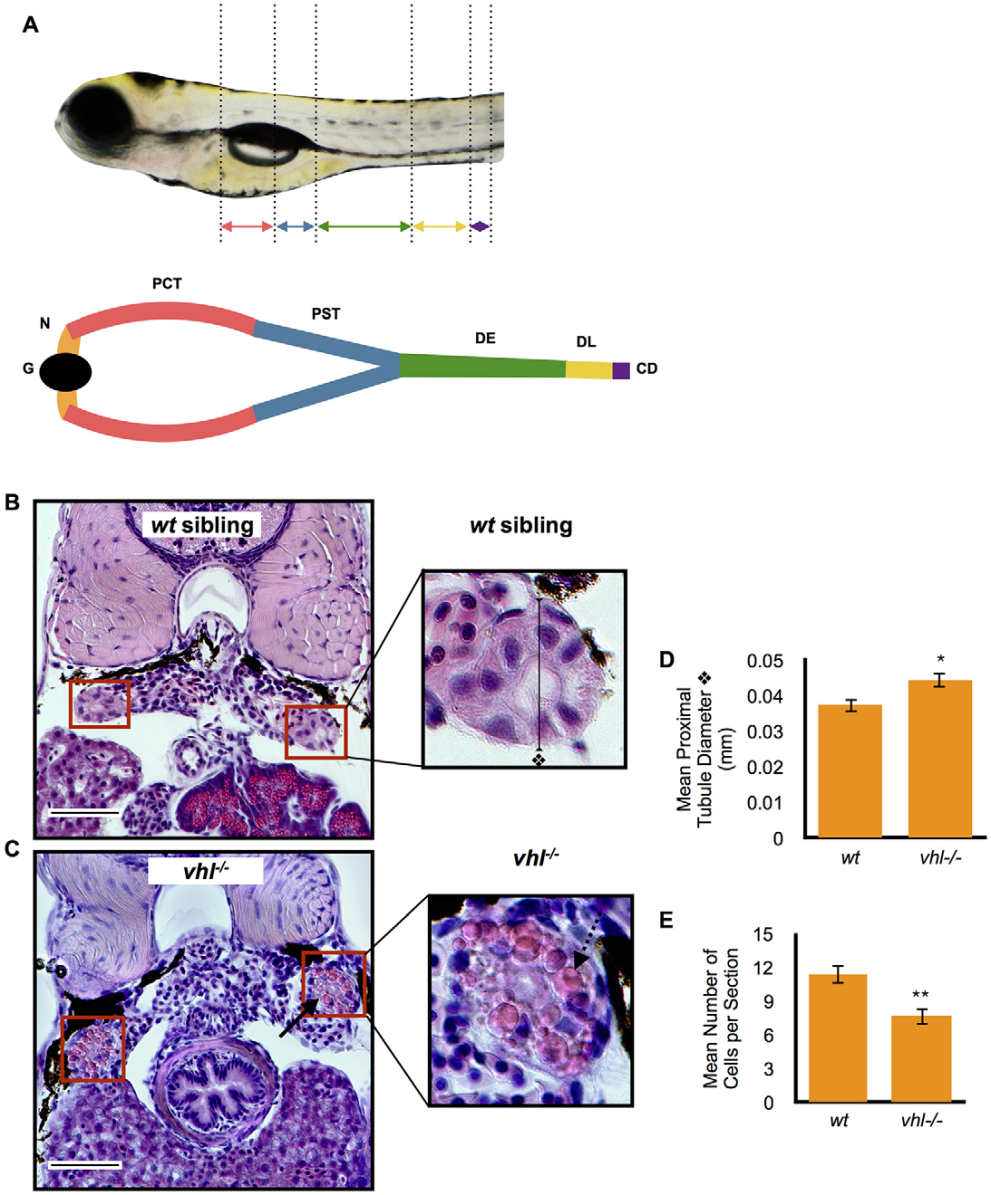
**Proximal *vhl^−/−^* pronephric tubules have distorted architecture and contain cytoplasmic vesicles when compared to *wt* siblings.** (A) Location of the proximal and distal pronephric tubules in the zebrafish larval pronephric kidney. Kidney segments are color coded. The glomerulus (G, black), neck (N, orange), proximal convoluted tubule (PCT, red), proximal straight tubule (PST, blue), distal early (DE, green), distal late (DL, yellow), collecting duct (CD, purple) are highlighted. (B,C) 5.5 dpf *vhl^−/−^* and *wt* siblings were fixed and embedded in JB-4 and sectioned with a glass knife. H&E staining was performed and measurements were taken using ImageJ. H&E staining of *wt* sibling (B) and *vhl^−/−^* (C) proximal pronephric tubules (red boxes). Scale bars: 0.05 mm. (D) Diameter (mm) of proximal pronephric tubule in the transverse section. (E) Mean number of cells per transverse section. Each experiment had at least three larvae per sample group and was performed in duplicate. Data represent mean±s.e.m. *P<0.05, **P<0.01 (paired two-tailed *t-*test).

In order to develop a model of ccRCC in zebrafish, and considering that the first genetic event in the formation of this tumor is loss of VHL, we assayed *vhl*^−/−^ zebrafish pronephric epithelial cells for features of ccRCC cells. The zebrafish pronephros consists of a single glomerulus and only two nephrons, responsible for recovering necessary ions and small molecules before they are lost in the urine. The tubular epithelium of each nephron is divided into a proximal and a distal portion (Fig. 1A). The proximal pronephros can be further subdivided into two segments, the proximal convoluted tubule and the proximal straight tubule, whereas the distal pronephros is formed by the distal early and distal late segment. The proximal convoluted segment is analogous to the mammalian proximal tubule in regard to its histology and conserved absorptive function. The function of the proximal straight tubule is unclear (Drummond and Davidson, 2010). The most striking differences between the *vhl*^−/−^ larvae and their *wt* siblings were observed in the proximal, but not distal, pronephros. This corroborates the idea that ccRCC likely originates in the proximal tubule in humans (Rankin et al., 2006; Shuch et al., 2015; Haase, 2005; Chen et al., 2016).

### Proximal *vhl*^−/−^ pronephric tubules exhibit distorted architecture and contain cytoplasmic vesicles

To investigate the kidney structure in the *vhl* zebrafish model, we performed hematoxylin and eosin (H&E) staining on transverse histological sections of the proximal pronephric tubules in 5.5 days post fertilization (dpf) *vhl^−/−^* and *wt* sibling larvae. H&E staining immediately highlighted several striking differences between the *vhl^−/−^* zebrafish (Fig. 1C) and their *wt* siblings (Fig. 1B). Overall, the apical to basal polarity of the *vhl^−/−^* pronephric tubules appeared to be distorted compared to their *wt* siblings. The tubules showcased a significantly longer transversal diameter than their *wt* siblings (Fig. 1D) and their epithelial cells contained a prominent eosinophilic cytoplasm (black arrow in Fig. 1C) with fewer hematoxylin-stained nuclei per transverse section (Fig. 1E). In addition, the proximal *vhl^−/−^* pronephric epithelium contained abundant cytoplasmic vesicles (dotted arrow in Fig. 1C, magnified image) that were completely absent in the *wt* siblings (Fig. 1B). This phenotype appeared to be present in all of the *vhl^−/−^* larvae, and absent in all of the *wt* siblings tested.

### Cytoplasmic vesicles in proximal *vhl*^−/−^ pronephric tubules contain lipids

**Fig. 2.**
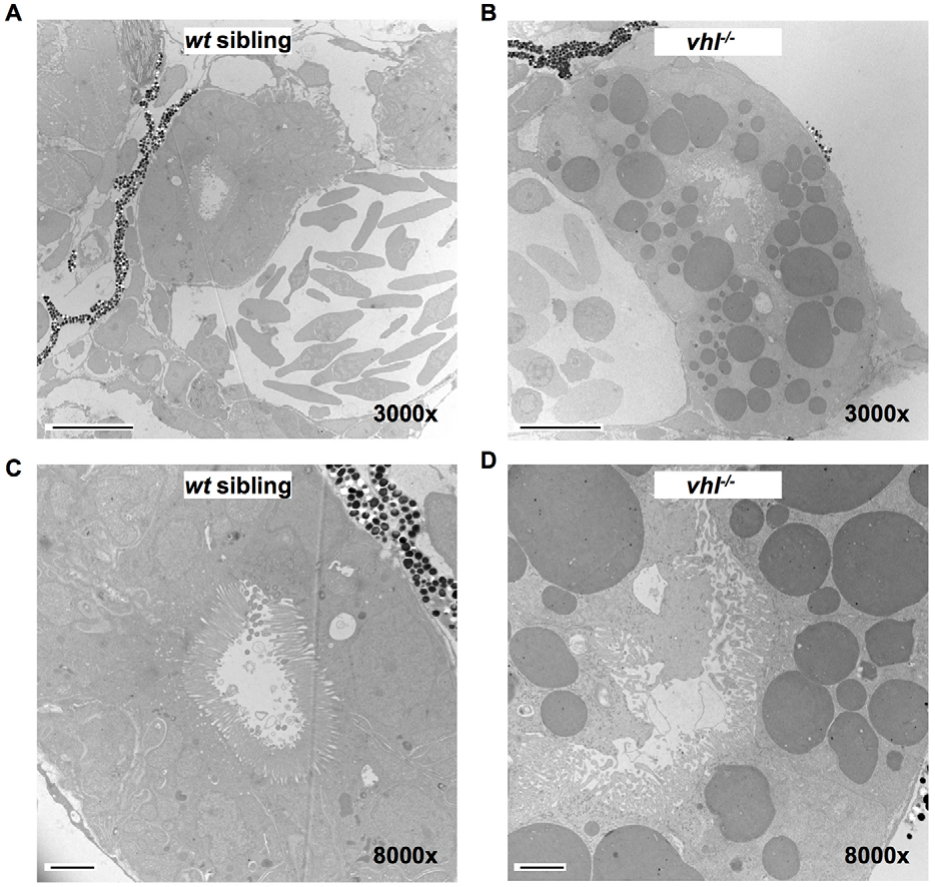
**TEM confirms that proximal *vhl^−/−^* pronephric tubules have an increased tubule diameter, a distorted lumen, disorganized cilia and contain cytoplasmic lipid vesicles.** 5.5 dpf *vhl^−/−^* and *wt* siblings were fixed, embedded and sectioned. Thin sections were stained with electron-dense metals and electron microscopy was performed. Transmission electron micrographs of proximal *vhl^−/−^* pronephros (B,D) in comparison to *wt* sibling (A,C)*.* Scale bars: 10 µm (A,B); 2 µm (C,D).

**Fig. 3.**
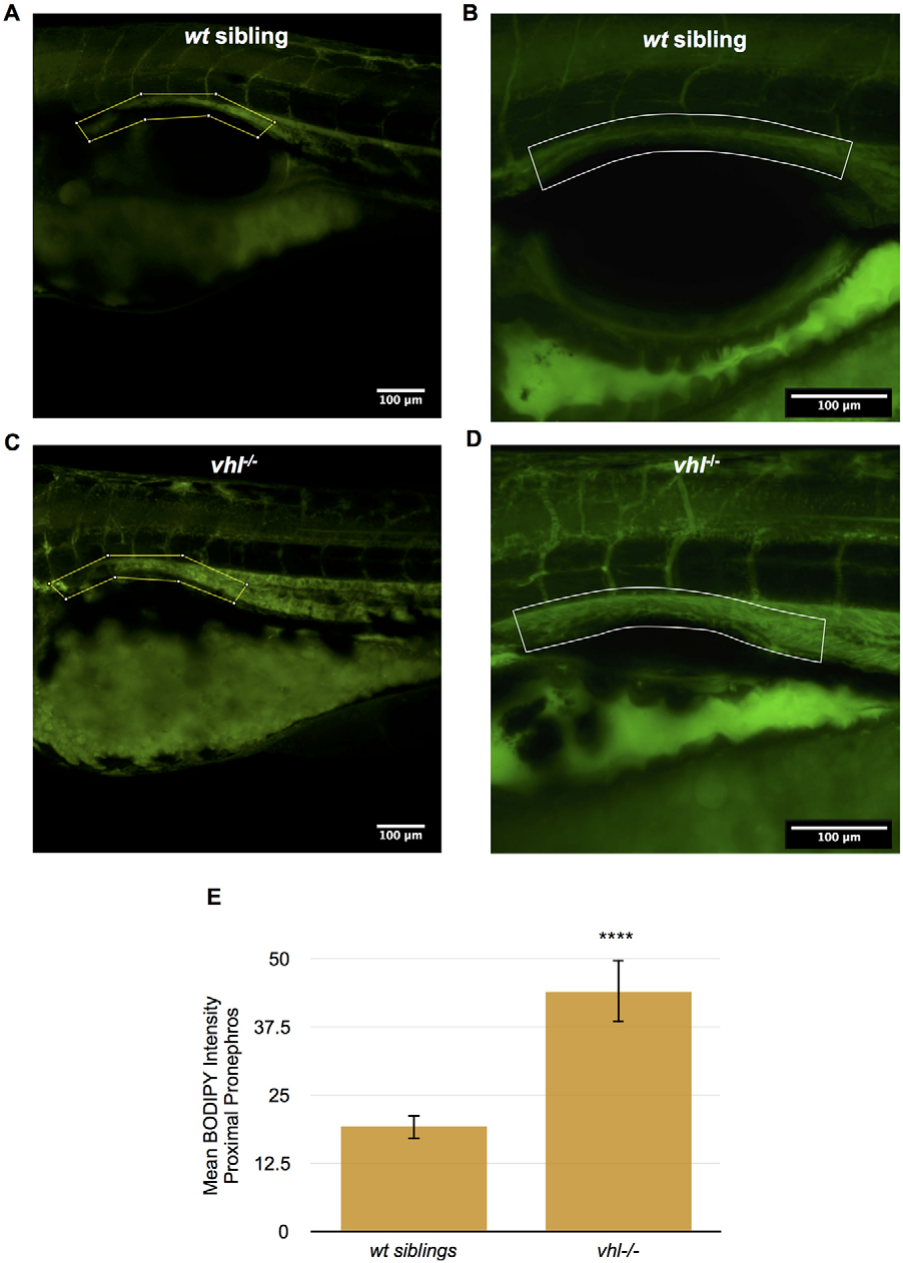
***Vhl^−/−^* larvae have an increased amount of lipids in the proximal pronephros compared to *wt* siblings.** (A-D) BODIPY 493/503 immunofluorescence was performed in live whole-mount 5.5 dpf *vhl^−/−^* and *wt* siblings. BODIPY 493/503 staining (in green) intensity was then quantified within a ROI encompassing the proximal pronephros (yellow box), using ImageJ. Confocal images of BODIPY staining in *vhl*^−/−^ (C) versus *wt* sibling (A). Confocal images of BODIPY staining in *vhl*^−/−^ (D) versus *wt* sibling (B). (E) Quantification of mean BODIPY 493/503 staining intensity within the ROI in 5.5 dpf *vhl^−/−^* versus *wt* siblings. Each experiment had at least three larvae per sample group and was performed in triplicate. Data represent mean±s.e.m. ****P<0.0001 (paired two-tailed *t-*test).

To further describe the structural abnormalities underlying the proximal *vhl^−/−^* pronephric epithelium, we performed transmission electron microscopy (TEM) on the pronephros of 5.5 dpf *vhl^−/−^* and their *wt* sibling larvae. Electron microscopy confirmed that the proximal *vhl^−/−^* pronephric tubule was enlarged compared to that of their *wt* siblings and displayed an extremely distorted lumen (Fig. 2A,B). Renal epithelium of *vhl^−/−^* larvae contained an abundance of darkly stained vesicles recognized in TEM images as lipid droplets (Fujimoto and Parton, 2011), resembling the clear cell histology of RCC. These lipid droplets were completely absent in the *wt* sibling proximal pronephric epithelium. At 8000× magnification, it also became apparent that the cilia coating the tubule lumen, which were neatly packed in the *wt* siblings, were disorganized in parts of the *vhl^−/−^* lumen (Fig. 2C,D). To confirm that the dark vesicles seen in TEM are in fact lipids, live *vhl^−/−^* and *wt* sibling larvae were stained with BODIPY 493/503, a lipophilic fluorescent dye used to quantify lipid content. *Vhl^−/−^* larvae exhibited increased BODIPY staining at 5.5 dpf in their proximal pronephric tubule compared to their *wt* siblings (Fig. 3A-D). To quantify the staining intensity within the proximal pronephric tubule only, we created a region of interest (ROI) in ImageJ surrounding the target area. When quantified, it became evident that the *vhl^−/−^* larvae contained significantly more lipids in the proximal pronephros than their *wt* siblings (Fig. 3E). The *vhl^−/−^* larvae also had significantly more BODIPY staining in their distal pronephros (Fig. S1A-C). Overall, the ratio of BODIPY staining between mutants and *wt* siblings is very similar in both the proximal and distal regions of the pronephros (Fig. S1D).

### Proximal *vhl*^−/−^ pronephric tubules contain glycogen

**Fig. 4.**
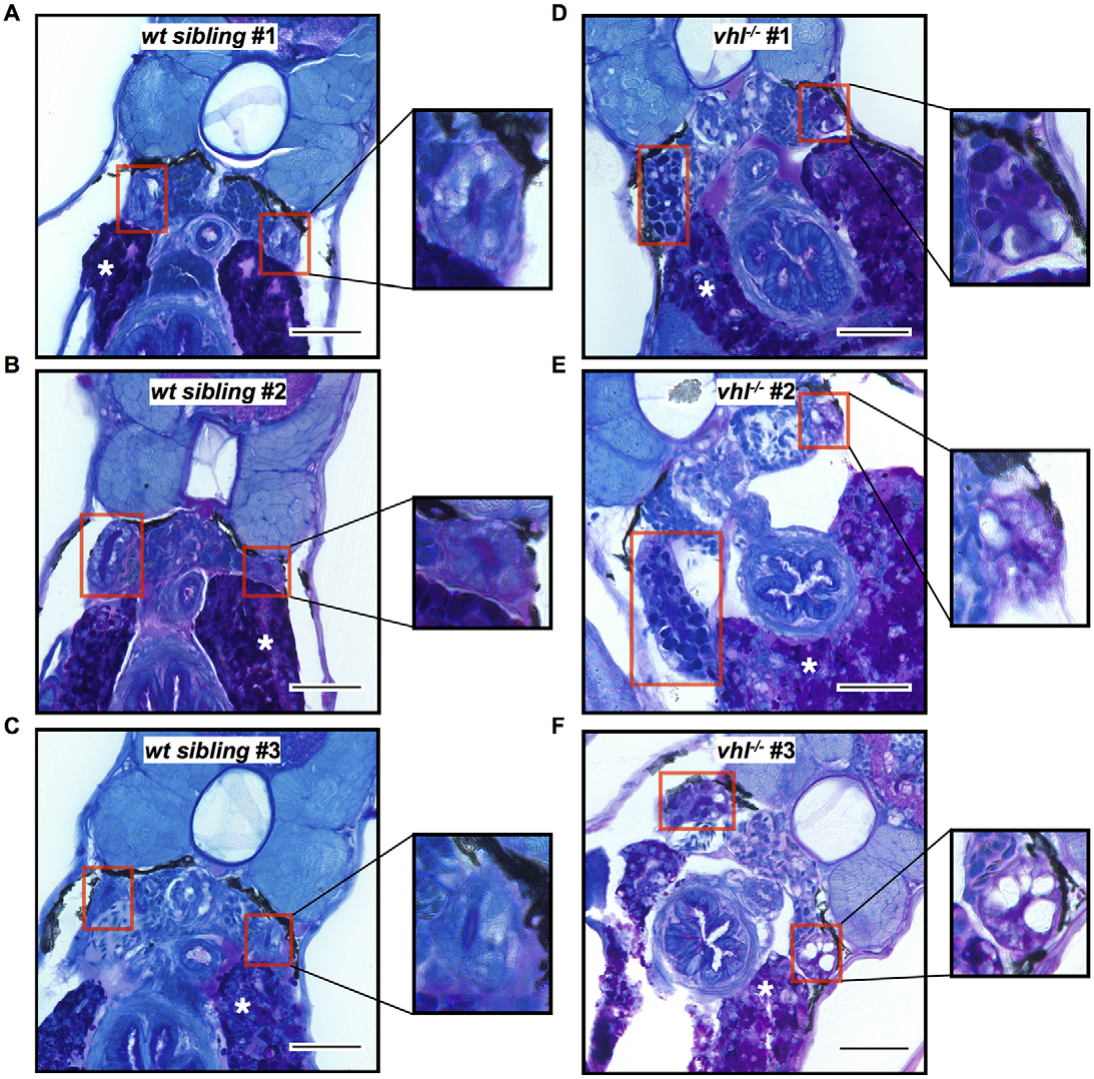
***Vhl*^−/−^** **larvae** **have increased glycogen content in the proximal pronephros compared to *wt* siblings.** 5.5 dpf *vhl^−/−^* and siblings were fixed and embedded in JB-4 and sectioned with a glass knife. PAS staining was performed with glycogen stained in purple. PAS staining of *wt* siblings (A-C) and *vhl^−/−^* (D-F) proximal pronephric tubules (red boxes). Asterisks indicate the liver, which serves as a positive control. Scale bars: 0.05 mm. Each experiment had at least five larvae per sample group and was performed in duplicate.

The presence of cellular glycogen is commonly associated with the clear cell histology observed in clear cell renal cell carcinoma (Shuch et al., 2015; Truong and Shen, 2011). To understand the impact of glycogen on the pronephros of 5.5 dpf *vhl^−/−^* and *wt* siblings, periodic acid Schiff (PAS) staining was performed. After this reaction, glycogen stains dark pink to purple, nuclei stain dark blue and cytoplasm light blue (Taksir et al., 2007). PAS staining confirmed that the proximal *vhl^−/−^* pronephric tubule (Fig. 4D-F) has increased glycogen compared to their *wt* siblings (Fig. 4A-C). The liver serves as a positive control for the PAS stain (indicated in each image with a white asterisk) given that it is known to accumulate glycogen until 6 dpf in unfed *wt* larvae (Pack et al., 1996).

### The proximal *vhl*^−/−^ pronephric tubule displays increased proliferation throughout development

**Fig. 5.**
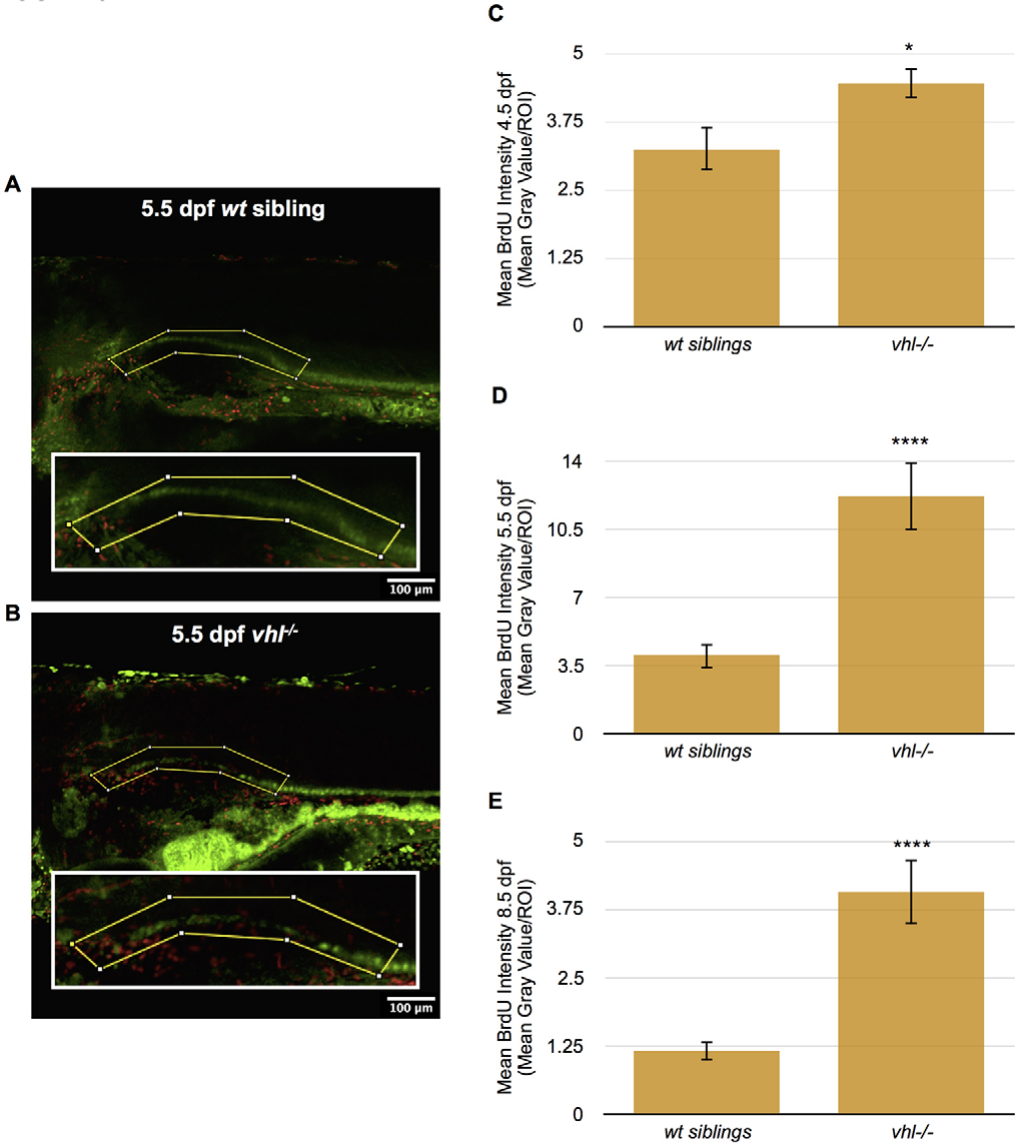
**Proximal *vhl^−/−^* pronephric tubules have increased cellular proliferation throughout development compared to *wt* siblings.** (A,B) BrdU immunofluorescence was performed in whole-mount 4.5, 5.5 and 8.5 dpf *Tg(ATPase1.a1A4:GFP) vhl^−/−^* and *wt* siblings. BrdU staining intensity (red) was then quantified within a ROI (yellow box) encompassing the proximal pronephros (green) using ImageJ. BrdU staining in 5.5 dpf *wt* sibling (A) and *vhl*^−/−^ (B). (C) Quantification of mean BrdU staining intensity within a ROI in 4.5 dpf *Tg(ATPase1.a1A4:GFP) vhl^−/−^* versus *wt* siblings. (D) Quantification of mean BrdU staining intensity within a ROI in 5.5 dpf *Tg(ATPase1.a1A4:GFP) vhl^−/−^* versus *wt* siblings. (E) Quantification of mean BrdU staining intensity within a ROI in 8.5 dpf *Tg(ATPase1.a1A4:GFP) vhl^−/−^* versus *wt* siblings. Each 4.5 dpf experiment had at least nine larvae per sample group and was performed in duplicate. Each 5.5 dpf experiment had on average ten larvae per sample group and was performed four times. Each 8.5 dpf experiment had 11 larvae per sample group and was performed in duplicate. Data represent mean±s.e.m. *P<0.05, ****P<0.0001 (paired two-tailed *t-*test).

RCC in humans is characterized by the presence of highly proliferative tumor cells (Schraml et al., 2009). To investigate whether the pronephros of *vhl*^−/−^ zebrafish larvae recapitulates this feature of human ccRCC, we stained 4.5, 5.5 and 8.5 dpf *vhl^−/−^* and *wt* sibling larvae with BrdU, a nucleoside analog which is incorporated into DNA during the S phase and therefore indicates cell proliferation. For these studies, we used a transgenic zebrafish line, Tg(ATPase1.a1A4:GFP), in which the pronephric tubule and duct are marked by GFP expression driven by the Na^+^/K^+^ ATPase promoter (Majumdar et al., 2000). We performed confocal microscopy using these transgenic lines to identify the proximal pronephros in the larvae and quantified the BrdU intensity in this specific region. For this purpose, we created a ROI surrounding the proximal pronephros, using ImageJ (Fig. 5A,B). *Vhl^−/−^* larval pronephric epithelium displayed significantly higher cellular proliferation compared to *wt* siblings starting at 4.5 dpf and continuing through 5.5 and 8.5 dpf (Fig. 5C-E).

### The proximal *vhl*^−/−^ pronephric tubule displays increased apoptosis in early development

**Fig. 6.**
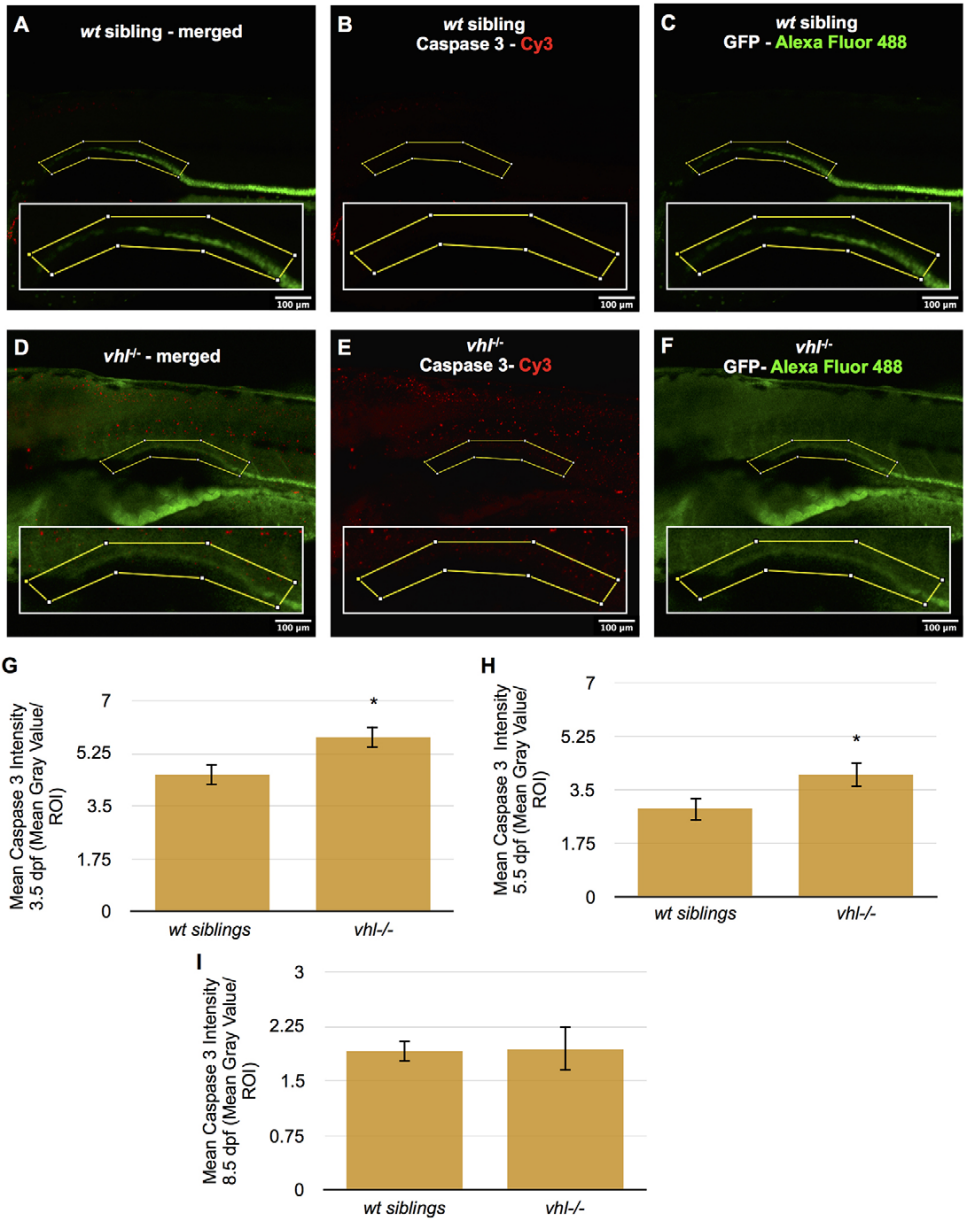
**Proximal *vhl^−/−^* pronephric tubules have increased cell death at 3.5 and 5.5 dpf compared to *wt* siblings.** (A-F) Caspase 3 immunofluorescence was performed in whole-mount 3.5, 5.5 and 8.5 dpf *Tg(ATPase1.a1A4:GFP) vhl^−/−^* and *wt* siblings. Caspase 3 staining intensity (red) was then quantified within an ROI (yellow box) encompassing the proximal pronephros (green) using ImageJ. Merged caspase 3 (red) and GFP (green) staining in 5.5 dpf *wt* sibling (A) and *vhl*^−/−^ (D) larvae. Caspase 3 staining only is shown in 5.5 dpf *wt* sibling (B) and *vhl*^−/−^ (E). GFP staining only is shown in 5.5 dpf *wt* sibling (C) and *vhl*^−/−^ (F). (G) Quantification of the mean caspase 3 staining intensity within a ROI in 3.5 dpf *vhl*^−/−^ versus *wt* siblings, (H) 5.5 dpf *vhl*^−/−^ versus *wt* siblings and (I) 8.5 dpf *vhl*^−/−^ versus *wt* siblings. To assist with visualization of caspase 3 staining, the contrast of this image has been adjusted globally using ImageJ software. The quantitative analysis presented was performed on non-enhanced images. Each 3.5, 5.5 and 8.5 dpf experiment had ten larvae per sample group and was performed in duplicate. Data represent mean±s.e.m. *P<0.05 (paired two-tailed *t-*test).

In order to examine the conflicting observations of increased cellular proliferation (Fig. 5) and fewer cells per transverse section (Fig. 1E) in the proximal tubule of 5.5 dpf *vhl^−/−^* larvae, we quantified the number of apoptotic cells in the region by immunofluorescence for activated caspase 3 (Sorrells et al., 2013; Tait and Green, 2010; Taylor et al., 2008). The caspase 3 assay was performed in whole-mount *Tg(ATPase1.a1A4:GFP) vhl^−/−^* and *wt* siblings at 3.5, 5.5 and 8.5 dpf. We performed confocal microscopy and quantified the caspase 3 intensity within a ROI surrounding the proximal pronephros (Fig. 6A-F). *Vhl^−/−^* larval pronephric epithelium displayed significantly higher cell death compared to *wt* siblings at 3.5 and 5.5 dpf. This difference was not detected at 8.5 dpf (Fig. 6G-I).

### Treatment with the HIF2a inhibitor Compound 76 partially rescues proximal pronephric tubule disorganization in the *vhl*^−/−^ larvae

**Fig. 7.**
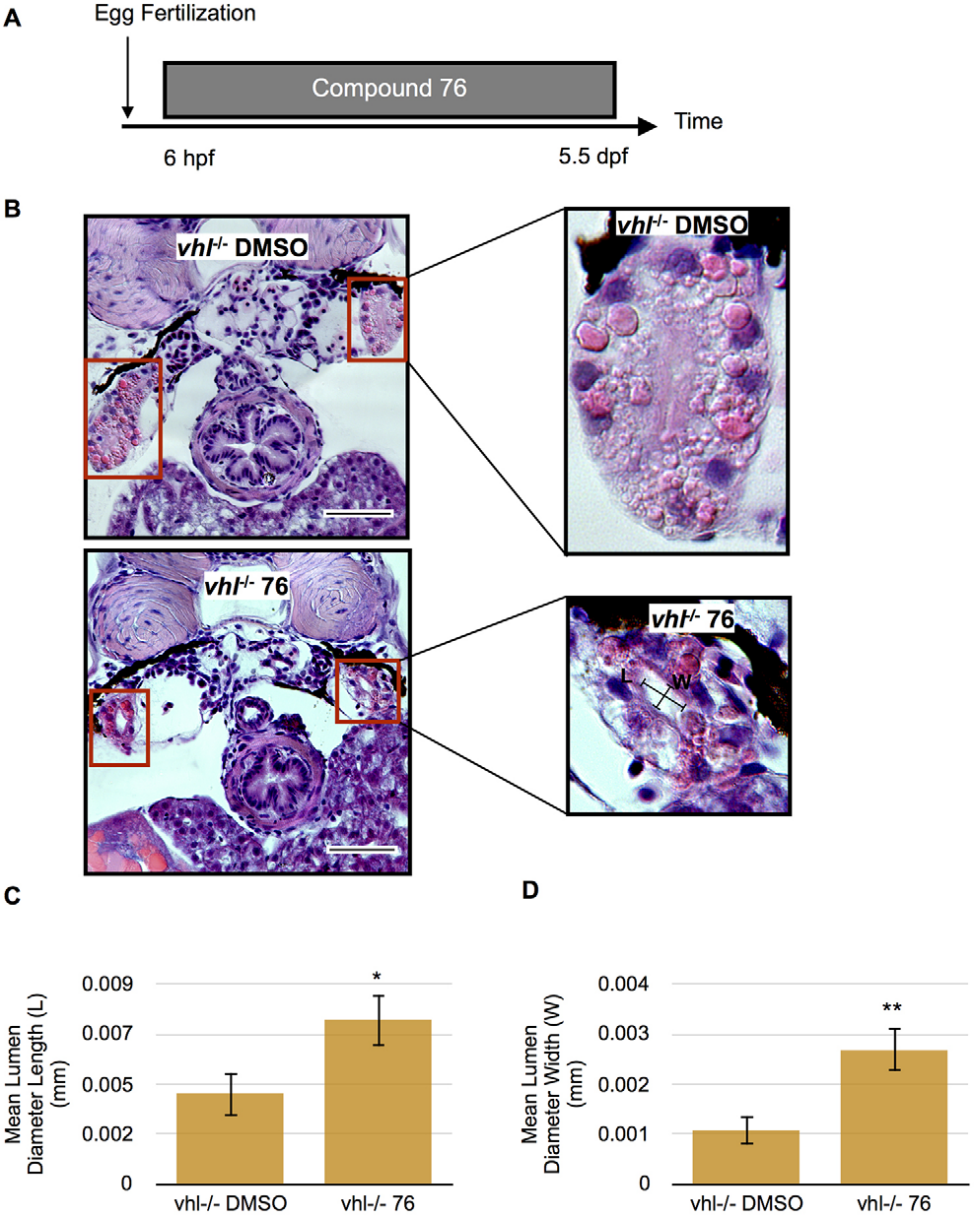
**Proximal *vhl^−/−^* pronephric tubules exhibit improved phenotype when treated with HIF2a inhibitor Compound 76.** (A) Treatment protocol. *Vhl*^−/−^ embryos were treated with 1 µM Compound 76 (76) or DMSO vehicle control from 6 hpf to 5.5 dpf. (B) H&E staining of 5.5 dpf *vhl*^−/−^ proximal pronephric tubules (red boxes) treated with Compound 76 compared to *vhl*^−/−^ treated with DMSO vehicle control. Scale bars: 0.05 mm. (C) Quantification of mean lumen diameter length (transverse section) in 5.5 dpf *vhl*^−/−^ larvae treated with Compound 76 or DMSO control. (D) Quantification of mean lumen diameter width (transverse section) in 5.5 dpf *vhl*^−/−^ treated with Compound 76 or DMSO control. Each experiment had at least five larvae per sample group and was performed in duplicate. Data represent mean±s.e.m. *P<0.05, **P<0.01 (paired two-tailed *t-*test).

To test whether expression of the zebrafish orthologs of human HIF2a are responsible for the kidney phenotype observed in the *vhl^−/−^* larvae, we treated the *vhl^−/−^* and *wt* sibling larvae with 1 µM Compound 76 or DMSO vehicle control from 6 hpf to 5.5 dpf (Fig. 7A). Compound 76 is a specific inhibitor of HIF2a that is active in mammalian cells and in zebrafish (Zimmer et al., 2008; Metelo et al., 2015). Treatment of *vhl^−/−^* larvae with Compound 76 significantly improved the proximal pronephric tubule phenotype compared to *vhl^−/−^* larvae treated only with DMSO (Fig. 7B). Compound-76-treated fish had more organized nuclei, fewer lipid vesicles and a significantly larger and more structured lumen (seen in transverse sections) (Fig. 7C,D)*.* To ensure that the activity of Compound 76 was indeed mediated by Hif2a inhibition instead of nonspecific binding of the small molecule, we treated larvae with the inactive Hif2a inhibitor Compound 05. Owing to some toxicity of Compound 05 at 1 µM concentration, compound treatments were performed from 2 to 5.5 dpf (Fig. S2A). Despite the reduction in treatment duration, compound 76 once again partially rescued the proximal pronephros phenotype with more organized cell architecture, a decrease in lipid vesicles, and a significantly larger lumen length and width (Fig. S2B-D). The rescue of the phenotype by compound treatment is not a mere result of an effect of the drug on development; the latter is not affected by compound treatment (Fig. S2B,E).

## DISCUSSION

We identify here for the first time a zebrafish model of early stage human ccRCC lesions. Based on histology, the *vhl^−/−^* mutant larvae exhibit disorganization in their proximal pronephric tubules. Specifically, they have a larger proximal tubule diameter and a diminished number of cells per transverse section. The mutant proximal pronephric tubules also contain lipid-filled cytoplasmic vesicles, aberrant glycogen accumulation, a distorted lumen and disorderly cilia compared to their *wt* siblings. The *vhl^−/−^* mutants display abnormal cell proliferation throughout their development as well as early-stage apoptosis along their proximal pronephric tubule when compared with their siblings. Finally, when treated with the HIF2a inhibitor, Compound 76, the abnormal structure of the proximal *vhl^−/−^* pronephric tubule is partially rescued.

The origin of human ccRCC from the proximal and/or distal nephron has been a matter of debate. Early morphological studies have supported the notion that human ccRCC expresses markers of both the distal and proximal nephron (Cohen et al., 1988; Hughson et al., 1993; Droz et al., 1990). Since then, Mandriota et al. have reported that in VHL patients, multicellular lesions were mostly derived from the segments expressing Tamm–Horsfall protein, which include the medullary thick ascending loop of Henle and the early distal nephron, whereas single cell malignant foci were most frequently found in the proximal tubular cells (Mandriota et al., 2002; Haase, 2005). Recent molecular profiling of the ‘classic’ histologic subtypes of kidney cancers supports the hypothesis that the molecular markers of ccRCC align more closely with the molecular signature of cells micro-dissected from the proximal tubular epithelium (Chen et al., 2016; Cheval et al., 2012; Davis et al., 2014). Our analysis of the *vhl*^−/−^ larval pronephros, although it cannot be extrapolated directly to human ccRCC, supports the notion that the proximal nephron is a preferred nidus of abnormal proliferation and likely an origin of ccRCC upon loss of *VHL*.

Human ccRCC is characterized by cells with eosinophilic cytoplasm and an abundant accumulation of lipid droplets and glycogen, providing cells with the characteristic clear appearance following fixation (Shuch et al., 2015; Truong and Shen, 2011). In hypoxia, cells adapt to consume less oxygen by shifting energy production from mitochondrial fatty acid β-oxidation to glycolysis. HIF1a inhibits β-oxidation by repressing the expression of medium and long-chain acyl-CoA dehydrogenases that catalyze the first step of β-oxidation. This metabolic reprogramming to limit acetyl-CoA generation by fatty acid β-oxidation, and therefore tricarboxylic acid cycle activity, leads to lipid accumulation and is characteristic of hypoxic cells and advanced cancers (Huang et al., 2014). In addition, HIF2a promotes the expression of perilipin 2, a lipid-droplet-coated protein, which is overexpressed in human ccRCC tumors and allows the formation of lipid droplets (Qiu et al., 2015). Therefore, in the absence of pVHL, the stabilization of HIF1a and HIF2a leads to lipid accumulation in the cell. As a result, we would expect the *vhl^−/−^* mutants which uniformly lack VHL protein to contain more fat. Our transmission electron microscopy results indeed indicate that the cytoplasmic vesicles seen in the proximal *vhl^−/−^* pronephric tubules contain lipids.

The presence of lipids is confirmed by BODIPY staining, which indicates that there is an overall increase in lipid accumulation in the *vhl*^−/−^ mutant fish compared to their *wt* siblings. BODIPY 493/503 fluorescent dye is specifically used to stain neutral lipids that accumulate in lipid droplets (Gocze and Freeman, 1994). Neutral lipids have been found to accumulate in *Vhl*-deficient hepatocytes resulting in the clear cell morphology characteristic of ccRCC (Haase et al., 2001). In addition to triglycerides, it has been found that cholesteryl esters also accumulate in clear cell renal cell carcinoma (Gebhard et al., 1987). Sphingolipid content has been found to change in aggressive forms of ccRCC (Sito et al., 2006). Further exploration using available BODIPY probes and lipidomic analysis is required to evaluate the effect of pVHL loss in our mutant fish on cholesteryl esters and other lipid types. Compared to their *wt* siblings, the *vhl^−/−^* mutants have significantly more lipids in their proximal pronephros. The same is seen in the distal pronephros resulting from an overall trend towards lipid accumulation in the entire *vhl^−/−^* mutant larvae. The ratio of lipid staining between *vhl^−/−^* mutant and *wt* siblings is comparable in both the proximal and distal pronephros, which indicates that the degree of lipid accumulation is similar throughout the entire *vhl^−/−^* pronephros.

The clear cell morphology of ccRCC refers to the presence of clear cytoplasm resulting from lipid or glycogen fixation artifacts (Shuch et al., 2015; Truong and Shen, 2011). Our TEM and BODIPY results confirm the presence of lipids in the proximal *vhl^−/−^* nephron, and our PAS staining results suggest the involvement of glycogen as well. An example of this classic clear cell histology is nicely seen in the *vhl^−/−^* mutant pronephros in Fig. 4F. The majority of the proximal *vhl^−/−^* kidneys stain purple with PAS, demonstrating the presence of glycogen, whereas almost all of the *wt* siblings stain entirely blue, signifying a lack of glycogen. Eosinophilic cytoplasm is another hallmark of ccRCC histology (Truong and Shen, 2011). The proximal *vhl^−/−^* pronephros, in addition to presenting cytoplasmic lipid vesicles, as described above, also displays expanded eosin (pink) staining compared to *wt* siblings. The histology of the *vhl^−/−^* mutant pronephros is very similar to human ccRCC, therefore we believe it represents an example of early stage ccRCC tumor formation. However, considering that the *vhl^−/−^* mutants die around 13 dpf, we cannot monitor the progression of tumor formation beyond this point. The cause of this early lethality is mostly unknown, although the development of a large pericardial edema and impaired cardiac contractility might contribute.

Rankin et al. developed a mouse model of VHL-associated renal disease, using the phosphoenolpyruvate carboxykinase (PEPCK) promoter to generate a mouse with Cre-recombinase expression in the proximal renal tubule (Rankin et al., 2006). They found that conditional inactivation of *Vhl* in these PEPCK-Cre mutants caused the development of both macroscopic and microscopic renal cysts, but not ccRCC. These tubular microcysts morphologically resembled renal cysts found in VHL patients, with eosinophilic cuboidal epithelium that appeared benign but without any evidence of nuclear abnormalities. Although mice in which murine *Vhl* has been inactivated in the proximal tubule (PEPCK-Vhlh) do not develop ccRCC, possibly because multiple genetic events (in addition to the loss of VHL) are required for full ccRCC development, their renal tubule cells show increased proliferation measured by Ki-67. Our *vhl^−/−^* zebrafish model mimics some aspects of the tubular microcysts described in this mouse, including eosinophilic epithelium and cell proliferation. ccRCC is typically a proliferative tumor (Schraml et al., 2009). Our data from BrdU staining, indicates that the proximal *vhl^−/−^* pronephric tubules exhibit a significantly greater amount of cell proliferation, spanning development, when compared to their *wt* siblings. Cell death, observed in earlier stages of development, offers an explanation for the appearance of cellular proliferation in *vhl^−/−^* larvae with fewer nuclei per section.

The maintenance of the primary cilia is one of the known HIF-independent functions of pVHL (Schraml et al., 2009). Cilia are composed of microtubules and are thought to be important in cell cycle control. pVHL binds to the cellular microtubules and stabilizes them by protecting from depolymerization. This microtubule stabilization is required for cilium formation. pVHL is located in the primary cilium of both human and mouse kidney cells (Schraml et al., 2009). Defects in primary cilia have been linked to development of polycystic disease in the kidney, which also occurs in some VHL patients (Browne et al., 1997). In humans, epithelial cells of the nephron have cilia that uniformly extend into the tubule lumen (Schraml et al., 2009). VHL-associated ccRCC is characterized by very low frequencies of primary cilia compared with normal kidney tubules, whereas VHL-independent papillary RCCs exhibit a significantly higher frequency of ciliated cells (Schraml et al., 2009). Uniform cilia can be seen in the lumen of the proximal pronephric tubule in the *wt* sibling zebrafish*.* By contrast, the *vhl^−/−^* mutants discussed here exhibit lower frequencies of cilia in their proximal pronephric tubules compared to their *wt* siblings, resembling the phenotype previously found in ccRCC. In addition, we see cell proliferation in the *vhl^−/−^* tubules, similar to that described in human kidney epithelial cells when cilia structure is disturbed (Schraml et al., 2009).

Because HIF2a functions as an oncogene in VHL-associated and sporadic cases of ccRCC, we treated the *vhl^−/−^* mutants with a small-molecule HIF2a inhibitor, Compound 76 (Zimmer et al., 2008; Metelo et al., 2015). Compound-76-treated *vhl^−/−^* larvae exhibit a partially recovered proximal pronephros phenotype with more organized nuclei, fewer lipid vesicles, and a significantly larger and more structured lumen. This improvement in phenotype was detected when compound treatment began at both 6 hpf and 2 dpf. No difference was observed when *vhl^−/−^* larvae were treated with inactive Hif2a inhibitor Compound 05 or among the *wt* controls. Images of the overall morphology of compound-treated fish rule out developmental delay as the reason for recovery. We previously reported that during chronic hypoxia and early development of *vhl*^−/−^ zebrafish, *epas1a* is the main gene to be induced among the Hif paralogs (Metelo et al., 2015). In addition, we showed that both the zebrafish HIF2a orthologs, *epas1a* and *epas1b*, but not the HIF1a orthologs, are necessary to mediate the hypoxic upregulation of the Hif2a target genes *epo* and *vegfab* (Metelo et al., 2015)*.* Compound 76 has been shown to specifically bind and suppress signaling of both *epas1a* and *epas1b* but not the zebrafish HIF1a orthologs (Metelo et al., 2015). Taking all these data together, our compound treatment results strongly indicate that the *vhl*^−/−^ mutant pronephric tubule disorganization is driven, at least in part, by the zebrafish orthologs of human HIF2a. Direct genetic testing of this hypothesis will require genetic deletion of *epas* and *hif* paralogs in *vhl*^−/−^ embryos.

It has been shown that Hif2a is involved in hepatic outgrowth as well as in the expansion of the intestine and exocrine pancreas in zebrafish embryos (Lin et al., 2014). The recovery of the *vhl*^−/−^ pronephric abnormalities with Hif2a inhibitor Compound 76 indicates that Hif2a might also be important in the development of the zebrafish pronephros, but further study is required. The results of this drug test indicate that small-molecule HIF2a inhibitors show promise in ccRCC treatment and merit further exploration. Given the conservation of hypoxia, angiogenesis and erythropoiesis pathways between humans and fish (Kajimura et al., 2006; Rojas et al., 2007; Paffett-Lugassy et al., 2007), zebrafish *vhl* mutants provide a uniquely effective model for further studies of VHL-related tumor biology and ccRCC development, as well as a platform for developing innovative new drug treatments (MacRae and Peterson, 2015).

## MATERIALS AND METHODS

### Zebrafish strains

Zebrafish (*Danio rerio*) embryos were grown in the dark at 28.5°C in Tübingen E3 HEPES buffer. Animal experiments were conducted based on standard fish husbandry protocols according to US national guidelines. All animal experiments were approved by the Massachusetts General Hospital Subcommittee on Research Animal Care (OLAW assurance no. A3596-01).

*Vhl^hu2117^* fish were provided by Fredericus van Eeden (The University of Sheffield, Western Bank, Sheffield, UK). *Vhl*^−/−^ embryos were obtained from breeding pairs of *vhl^hu2117^*^+/−^ adult fish, and their genotype was confirmed by PCR using the following primers that span the mutation site (5′-CGTTGAAGCTTTAGTCTAACTCGG-3′ and 5′-CGAACCCACAAAAGTTGTTATTCT-3′). *Vhl*^−/−^ embryos and siblings (*wt* and *vhl*^+/−^ embryos) were divided at 3 dpf according to phenotypic differences.

*Tg(ATPase1.a1A4:GFP)* breeding pairs were provided by Iain Drummond (Massachusetts General Hospital Division of Nephrology, Boston, MA). Heterozygous *vhl^hu2117^*^+/−^ mutants were crossed with the transgenic line *Tg(ATPase1.a1A4:GFP)* in order to obtain *Tg(ATPase1.a1A4:GFP)(vhl*^+/+^*)* and *Tg(ATPase1.a1A4:GFP)(vhl*^+/−^*)* adults. *Tg(ATPase1.a1A4:GFP)(vhl*^+/−^*)* were crossed to obtain *Tg(ATPase1.a1A4:GFP)(vhl*^−/−^*)* embryos. *Vhl*^−/−^ embryos and siblings (*wt* and *vhl*^+/−^ embryos) were divided at 3 dpf according to phenotypic differences.

### Histological sectioning and hematoxylin and eosin staining

5.5 dpf *vhl^−/−^* and *wt* siblings were fixed in 4% paraformaldehyde overnight at 4°C. Specimens were then dehydrated using increasing concentrations of ethanol to 100%, followed by 100% methanol. Larvae were then infiltrated, using catalyzed JB-4™ solution, and embedded according to manufacturer's instructions (JB-4 Embedding Kit; cat. #00226, Polysciences). The solidified JB-4™ block was trimmed and mounted to a plastic block holder. Samples were sectioned transversally using a glass knife at a thickness of 7 µm, on a Leica RM 2165 microtome, and placed on Superfrost Plus microscope slides (Fisherbrand #12-550-15). H&E staining was performed as previously described (Sullivan-Brown et al., 2011). Gill's #2 Hematoxylin was purchased from Sigma-Aldrich (cat. #GHS216). Eosin Y powder was purchased from Polysciences (cat. #02740) and dissolved as described previously (Sullivan-Brown et al., 2011). Images were taken on a Nikon Eclipse E800 microscope at 60× magnification. Proximal pronephric tubule measurements were calculated using ImageJ (http://imagej.nih.gov/ij/). The experiments shown in Fig. 1 had a minimum of three larvae per sample and were performed in duplicate. Tubule diameter was measured in transverse sections across the lumen in the proximal pronephros (Fig. 1B, magnified image). Lumen diameter length and width were also measured in transverse sections (Fig. 7B, lower magnified image).

### Transmission electron microscopy

5.5 dpf *vhl^−/−^* and *wt* siblings were fixed in 1.0% paraformaldehyde and 1.5% glutaraldehyde in 3% sucrose, in 0.1 M sodium cacodylate buffer, pH 7.4 (Electron Microscopy Sciences, Hatfield, PA) for at least 24 h at 4°C. Samples were rinsed in 0.1 M sodium cacodylate buffer, and post-fixed in 1.0% osmium tetroxide in cacodylate buffer for 1 h at room temperature. Samples were rinsed again in buffer, dehydrated through a graded series of ethanol solutions to 100%, dehydrated briefly in 100% propylene oxide and pre-infiltrated in a 1:1 mix of Eponate resin (Ted Pella, Redding, CA) and propylene oxide, overnight with gentle rotation. Specimens were then infiltrated with fresh Eponate resin for several hours, embedded in flat molds with fresh Eponate and allowed to polymerize overnight at 60°C. Thin sections were cut using a Leica EM UC7 ultramicrotome, collected onto formvar-coated grids, stained with uranyl acetate and lead citrate, and examined in a JEOL JEM 1011 transmission electron microscope at 80 kV. Images were collected using an AMT digital imaging system (Advanced Microscopy Techniques, Danvers, MA).

### BODIPY 493/503 staining

5.5 dpf *vhl*^−/−^ and *wt* sibling larvae were incubated in E3 medium containing 100 µM BODIPY 493/503 (4,4-Difluoro-1,3,5,7,8-Pentamethyl-4-Bora-3a,4a-Diaza-s-Indacene; Invitrogen #D-3922) in DMSO (1% final DMSO concentration), in the dark for 1 h at 28°C. Following incubation, larvae were washed three times with E3 buffer and anesthetized in 0.4% Tricaine. Larvae were mounted in 1% low-melting-point agarose, covered in E3 buffer, and imaged on a confocal microscope as described below. The protocol was adapted from Detrich et al. (2009). Each experiment had at least three larvae per sample group and was performed in triplicate.

### Periodic acid Schiff staining

5.5 dpf *vhl*^−/−^ and *wt* sibling larvae were fixed in 4% paraformaldehyde overnight at 4°C. After fixation, larvae were washed twice for 10 min in 0.2 mol/l sodium cacodylate buffer (pH 7.3). Sodium cacodylate trihydrate was purchased from Sigma (cat. #C0250-10G). Larvae were then post-fixed in a 1:1 mixture of 2% osmium tetroxide in 0.2 M cacodylate buffer and 5% potassium dichromate for 1 h, then rinsed five times for 5 min with distilled water until clear. Osmium tetroxide was purchased from Sigma (cat. #201030-100MG), as was potassium dichromate (cat. #P5271-25G). Samples were then dehydrated, infiltrated, embedded and sectioned in JB-4™ as described above. Slides were hydrated in distilled water for 5 min, oxidized in 0.5% periodic acid solution (Polysciences #25874) for 10 min, and rinsed for 1 min in distilled water. 50% Schiff's reagent (Polysciences #25876-16) was applied for 20 min at room temperature. Slides were rinsed in running water for 7 min and dried at 45°C for 10 min. A filtered, 1:20 dilution of Richardson's stock solution [equal parts 1% Methylene Blue (Sigma #M9140-25G) in 1% borax (VWR #RC105016) and 1% azure II (Sigma #861065-25G)] was used to counterstain for 1 min and slides were then rinsed under running water for 5 min. Slides were dehydrated in 95% and 100% ethanol, and cleared in xylene twice, for 2 min each. Slides were coverslipped in Acrytol mounting media (Fisher #13518). The protocol was adapted from Taksir et al. (2007). Images were taken on a Nikon Eclipse E800 microscope at 60× magnification. Each experiment had a minimum of 5 larvae per sample and was performed in duplicate.

### 5-Bromo-2**′**-deoxyuridine staining

*Tg(ATPase1.a1A4:GFP)* *(vhl^−/−^)* zebrafish and siblings were treated at 3.5, 4.5 or 7.5 dpf with E3 medium containing 10 mM 5-Bromo-2′-deoxyuridine (BrdU) in DMSO (1% final DMSO concentration). BrdU solution was buffered to neutral pH with sodium bicarbonate. Larvae were incubated for 24 h at 28.5°C and fixed in 4% paraformaldehyde overnight at 4°C. 4.5 dpf larvae were digested with 15 µg ml^−1^ Proteinase K at 37°C for 50 min, 5.5 dpf samples for 55 min and 8.5 dpf samples for 80 min. Next, they were treated with 2 M HCl for 1 h and blocked in 10% goat serum [1% DMSO, 10% normal goat serum, 0.7% fish gelatin, PBS with 0.5% Tween-20 (0.5% PBST)] overnight at 4°C. Samples were washed with 0.1% PBST between steps. After blocking, specimens were washed 4× for 30 min in incubation solution (1% DMSO, 2% normal goat serum, 0.7% fish gelatin, 0.5% PBST) at room temperature. Primary antibodies were applied overnight at 4°C according to the following dilutions; 1:500 monoclonal anti-BrdU produced in mouse (Sigma #B3484) and 1:200 anti-GFP, rabbit serum (Life Technologies #A6455). Negative controls without primary antibodies were used to control for non-specific secondary antibody binding. Samples were washed 4× for 30 min in incubation solution at room temperature. Alexa-Fluor-546-conjugated goat anti-mouse-IgG (H+L) secondary antibody (Life Technologies #A11003) was applied at a 1:1000 dilution, along with Alexa-Fluor-488-conjugated goat anti-rabbit-IgG (H+L) secondary (Life Technologies #A11008) at a 1:1500 dilution. Samples were incubated overnight at 4°C. After washing larvae 4× for 30 min in incubation solution at room temperature, samples were stored in 0.5% PBST at 4°C until ready to image on a confocal microscope as described below. Each 4.5 dpf experiment had at least nine larvae per sample group and was performed in duplicate. Each 5.5 dpf experiment had on average ten larvae per sample group and was performed four times. Each 8.5 dpf experiment had 11 larvae per sample group and was performed in duplicate.

### Apoptotic assay for activated caspase 3 immunofluorescence

*Tg(ATPase1.a1A4:GFP)(vhl^−/−^)* 3.5, 5.5 and 8.5 dpf and siblings were fixed in 4% paraformaldehyde overnight at 4°C. Larvae were permeabilized with acetone for 7 min at −20°C and washed with 1× PDT (0.1% PBST, 0.3% Triton X-100, 1% DMSO) once. Then, samples were washed two times in fresh 1× PDT, rocking gently for 30 min each at room temperature. Larvae were immersed in blocking buffer (10% heat-inactivated FBS, 2% BSA, 1× 0.1% PBST) and rocked gently for 1 h at room temperature. Rabbit anti-active caspase-3 antibody (BD Pharmingen #559565) was applied at a 1:500 dilution and mouse anti-GFP antibody (Thermo Scientific #33-2600) at a 1:200 dilution. Negative controls did not receive any primary antibody. Samples were rocked gently overnight at 4°C and washed two times with 1× PDT the next morning. 1× PDT was added and larvae were rocked twice at room temperature for 30 min. Samples were rocked gently for 1 h at room temperature in blocking buffer. Cy3-conjugated AffiniPure donkey anti-rabbit-IgG (H+L) (Jackson ImmunoResearch Laboratories Inc. #711-165-152) (1:200) and Alexa-Fluor-488-conjugated goat anti-mouse-IgG (Invitrogen A21121) (1:1500) antibodies were added and samples gently rocked overnight at 4°C. Larvae were washed two times with 1× PDT. Fresh 1× PDT was added and samples were rocked at room temperature, two times for 30 min each. Fresh 1× PDT was added and samples were stored at 4°C for up to 48 h until ready to image on a confocal microscope as described below. The protocol was adapted from Sorrells et al. (2013). Each experiment had ten larvae per sample group and was performed in duplicate.

### Compound treatment

An entire clutch of *vhl*^−/−^ and *wt* sibling embryos was divided equally into three treatments, DMSO (0.01%), Compound 76 (1 µM in DMSO) or Compound 05 (1 µM in DMSO). Treatments were performed twice a day, from 6 hpf to 5.5 dpf or from 2 dpf to 5.5 dpf. At 5.5 dpf, the *vhl*^−/−^ larvae were separated from their *wt* and heterozygotic siblings based on phenotypic differences, and fixed in 4% paraformaldehyde overnight at 4°C. Specimens were dehydrated, embedded in JB-4, sectioned, and H&E stained as described above. Images were taken on a Nikon Eclipse E800 microscope at 60× magnification. Proximal pronephric tubule measurements were obtained using ImageJ. Each experiment had a minimum of five larvae per sample and was performed in duplicate.

### Confocal microscopy

Larvae were mounted in 1% low-melting-point agarose. Confocal images were taken on a Zeiss LSM 700 microscope using a 10× or 20× objective. Z-stack images were taken and processed using ImageJ. In the case of *Tg(ATPase1.a1A4:GFP)(vhl^+/−^* or *vhl^+/+^)* and *Tg(ATPase1.a1A4:GFP)(vhl^−/−^)*, only the images containing fluorescent pronephros were stacked. Using ImageJ, a ROI was selected surrounding the proximal or distal pronephros and used to calculate the mean intensity (mean gray value per ROI) for each *z*-stack.

### Statistics

Statistical analysis was performed for all experiments. Paired two-tailed *t-*tests were used for all of the analyses. Differences between two groups were considered significant when the *P* value was less than or equal to 0.05. The highest and lowest outlier was removed from Fig. 3E and Fig. S2C,D due to *in vivo* variation.
